# Customization of the angular spectrum method for calculating the acoustic piston field transmitted through a solid plate using MATLAB

**DOI:** 10.1016/j.mex.2023.102037

**Published:** 2023-01-24

**Authors:** M.M. Sæther

**Affiliations:** Department of Physics and Technology, University of Bergen, Bergen, Norway

**Keywords:** Angular spectrum model for acoustic wave propagation, Numerical integration involving an irregular oscillator, Open-source MATLAB program, Angular spectrum method

## Abstract

The angular spectrum (AS) model is customized to calculate the spatial acoustic pressure field generated by a piston source and transmitted through a steel plate immersed in water, for normal beam incidence. A MATLAB program is developed for this specific problem combining use of Gauss quadrature and a generalized Filon method. The program calculates the pressure wave number spectrum generated by the piston source and transforms the wave number spectrum into the spatial domain. Convergence analysis show that the MATLAB program is far more efficient than the more traditional approach of using the fast Fourier transform algorithm to transform the pressure wavenumber spectrum into the spatial domain. The MATLAB program is published here and free for others to use. The methods and MATLAB algorithms are obtained by

• Converting the original 2D AS model to a 1D model using cylindrical coordinates.
• Combining use of Gauss quadrature and a generalized Filon method for more accurate pressure calculations compared with use of the fast Fourier transform.
• Introducing adaptive numerical integration algorithms in MATLAB and error control parameters which are easy to use.

## Graphical abstract

**Figure:**
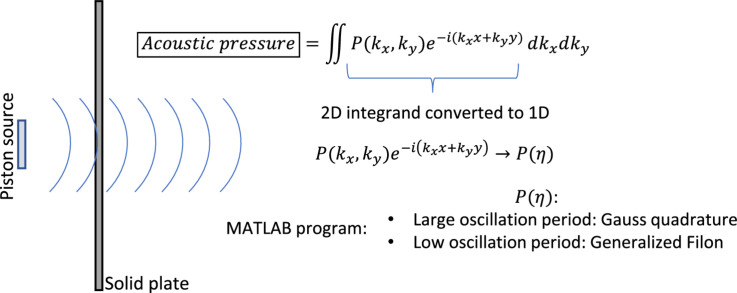

Specifications table

**Table:** 

Subject Area:	Computer Science
More specific subject area:	Mathematical numeric modeling
Method name:	Angular spectrum method
Name and reference of original method:	M. J. Anderson, P. R. Martin, and C. M. Fortunko, Resonant transmission of a three-dimensional acoustic sound beam through a solid plate in air: Theory and measurement, J. Acoust. Soc. Am. 98 (5) (1995) 2628–2638, https://doi.org/10.1121/1.413229.
Resource availability:	MATLAB code is included as supplementary material

## Background

The angular spectrum (AS) approach has been used for decades to simulate and study the interaction of ultrasound and a fluid-immersed solid plate [1], [2], [3], [4], [5], [6], [7], [8]. Anderson et al. [3] developed a model (ASM1) for beam transmission through such plates where a piston was used as the source. ASM1 is widely used but has some challenges related to high-accuracy pressure calculations [4]. The uniform grid used in the fast Fourier method typically used in the numerical calculations of ASM1 may give severe aliasing effects.

In the current work it is shown how these challenges may be resolved by customizing ASM1 into another model ASM2. A MATLAB code developed for the specific problem and free for others to use is presented here. The customized ASM2 has been used in [8] for high accuracy calculations of the transmitted pressure through a water-immersed steel plate at very large distances from the plate without the use of the MATLAB program presented here. As such, the current work also provides supplementary material to [8]. The MATLAB program is easy to use, has very few parameters for the user to specify and a detailed description of the program is given here. The program may be of use for others studying the interaction of ultrasound and solid plates. It is far more computationally efficient than implementations of ASM1 using the 2D fast Fourier transform algorithm, meaning that a higher accuracy is achieved using shorter computational time. The program provides accurate estimations of the pressure calculated at both long and short distances from the source. The ASM2 method with the current MATLAB implementation is especially well suited for studying near field effects which is proven to be present even at large distances from the source [8].

## Method details

In the following, the customization of ASM1 into ASM2 is first described. Next, the MATLAB program for ASM2 is presented in detail, and finally some convergence tests are given comparing the use of ASM1 and ASM2.

### Customization of the angular spectrum model

Anderson et al. [3] presented an AS model (ASM1) for beam transmission through a solid plate immersed in a fluid at normal beam incidence. The plate is assumed to be elastic, isotropic, homogeneous, and of infinite lateral extent. Using a time convention $e^{i\omega t}$ where ω=2πf is the angular frequency and *f* is the frequency, the transmitted pressure, *p_t_* is given as [3]:

(1) $$p_{t}\left(x,y,z,f\right)=\frac{e^{i\omega t}}{4\pi^{2}}\int_{-\infty }^{\infty }\int_{-\infty }^{\infty }\frac{\rho_{f}\omega }{h_{f,z}}HTe^{-ih_{f,z}\left(z-d\right)}e^{-i\left(h_{f,x}x+h_{f,y}y\right)}dh_{f,x}dh_{f,y}.$$

A cartesian coordinate system is used, with the front surface of the source located in the x-y plane at z = 0. $\rho_{f}$ is the fluid density, and *d* is the plate thickness. $h_{f,x}$, $h_{f,y}$, and $h_{f,z}$ are the x, y, and z components of the wave vector in the fluid, respectively, $h_{f}=\omega /c_{f}$ is the acoustic wavenumber in the fluid, where $c_{f}$ is the fluid sound velocity, and

(2) $$h_{f,z}=\left\{ \begin{array}{c} \sqrt{h_{f}^{2}-h_{f,x}^{2}-h_{f,y}^{2}},\;\;\;\;\;\;h_{f,x}^{2}+h_{f,y}^{2}\leq h_{f}^{2},\\ -i\sqrt{h_{f,x}^{2}+h_{f,y}^{2}-h_{f}^{2}\;},\;h_{f,x}^{2}+h_{f,y}^{2}>h_{f}^{2}. \end{array}\right.$$

For $h_{f,x}^{2}+h_{f,y}^{2}>h_{f}^{2}$, $h_{f,z}$ becomes imaginary, representing evanescent (exponentially decaying) waves in the z direction. T is the plane wave transmission coefficient and *H* is the aperture function which for a baffled piston is given as [3]

(3) $$H=2v_{0}\pi a^{2}\frac{J_{1}\left(a\sqrt{h_{f,x}^{2}+h_{f,y}^{2}}\right)}{a\sqrt{h_{f,x}^{2}+h_{f,y}^{2}}}.$$

*a* is the piston radius, *v_0_* is the particle velocity of the piston, and *J*_1_ is the first order Bessel function of the first kind. Note that in [3] the factor “2” in Eq. (3) was incorrectly left out. It has been shown in e.g. [7] that this factor must be included.

Eq. (1) (ASM1) may be regarded as a 2D Fourier transform which is typically solved using the 2D fast Fourier transform (FFT2) routine. However, due to sharp gradients in the integrand of Eq. (1), the uniform sampling grid used in the FFT2 routine may potentially cause large aliasing effects [4]. In the following it is shown how the 2D integral in Eq. (1) can be transformed into a 1D integral resulting in a new model (ASM2), which can be solved using an adaptive numerical integration model.

By coordinate transformation from Cartesian to cylindrical coordinates (r,φ,z), where $r=\sqrt{x^{2}+y^{2}}$ is the radial range and φ is the polar angle (in the *x-y* plane), Eq. (1) may be written as

(4) $$p_{t}\left(r,\varphi ,z,f\right)=\frac{e^{i\omega t}}{4\pi^{2}}\int_{0}^{2\pi }\int_{0}^{\infty }\frac{\rho_{f}\omega }{h_{f,z}}HTe^{-ih_{f,z}\left(z-d\right)}e^{-i\eta r\cos\varphi }\eta d\eta d\varphi .$$

η is the component of the acoustic wave vector in the plane of the plate (the r-direction), here referred to as the "horizontal wavenumber", and is expressed with $\eta^{2}=h_{f,x}^{2}+h_{f,y}^{2}$. By introducing the identity $J_{0}\left(\eta r\right)=\frac{1}{2\pi }\int_{0}^{2\pi }e^{-i\eta r\cos\varphi }d\varphi$ [8], and omitting the time dependent term $e^{i\omega t}$, Eq. (4) can be written as

(5) $$p_{t}\left(r,z,f\right)=\frac{1}{2\pi }\int_{0}^{\infty }\frac{\rho_{f}\omega }{h_{f,z}}HTe^{-ih_{f,z}\left(z-d\right)}J_{0}\left(\eta r\right)\eta d\eta .$$

For the incident pressure on the plate, *p_i_*, in absence of the plate,

(6) $$p_{i}\left(r,z_{0},f\right)=\frac{1}{2\pi }\int_{0}^{\infty }\frac{\rho_{f}\omega }{h_{f,z}}He^{-ih_{f,z}z_{0}}J_{0}\left(\eta r\right)\eta d\eta .$$

*z*_0_ is z-coordinate of the front surface of the plate. *J*_0_ is the zeroth order Bessel function of the first kind. Eqs. (5) and (6) represent the customized AS model ASM2 used in [8].

### MATLAB implementation of model

The integrands in Eqs. (5) and (6) go to zero for $\eta >h_{f}+\Delta \eta_{cutoff}$, and the integrals can be calculated numerically. The integrands have an irregular oscillator, which is a classical problem in numerical integration [9,10]. To numerically solve the integrals in Eqs. (5) and (6), two different numerical integration techniques are used: Gauss and the generalized Filon method, which are complimentary to each other. The generalized Filon method is given for integrals on the form [10]:

(7) $$I=\frac{1}{2\pi }\int_{\eta_{1}}^{\eta_{2}}f\left(\eta \right)e^{-ig\left(\eta \right)}d\eta .$$

Following [10], f(η) and g(η) are approximated by a second order polynomial, and the resulting integral is evaluated analytically. This method is suited for highly oscillatory integrands, which is not the case for the Gauss method. Visa versa, the Gauss method is suited for integrands without too much oscillations, which is not the case for the Filon method. The oscillation period is therefore tracked and the program chooses which of the Filon or Gauss method is to be used. The argument in the exponential in Eqs. (5) and (6) is nonlinear, but the instant oscillation period at a certain $\eta_{0}$ can be defined by a $\Delta \eta_{0}$ giving a 2π increase of the argument in the exponential, i.e.

(8) $$ih_{f,z}\left(\eta_{0}+\Delta \eta_{0}\right)z\;-\;ih_{f,z}\left(\eta_{0}+\Delta \eta_{0}\right)z=2\pi$$

By setting for example $\Delta \eta_{0}=0.2$ rad/m, $\eta_{0}$ for which the oscillation period is 0.2 rad/m is obtained, and $\eta_{0}$ is the value for which the Filon method is used instead of the Gauss method. The adaptive Gauss method is based on the 4^th^ and 5^th^ order Gauss quadrature rules. In the adaptive Filon method, Eq. (7) is calculated using first 3 and then 5 data points, producing a rough and a finer estimate of Eq. (7), and thus an error estimate.

In the MATLAB program, the Gauss method is used from η=0 and up to $\eta =\eta_{0}$. From $\eta =\eta_{0}$ and up to approximately $\eta =h_{f}$ the Filon method is used. For $\eta >h_{f}$ the argument is real giving no oscillation, and the Gauss method is used again. A practical way is used to determine the η for which the program switch from the Filon method and back to the Gauss method again. For η close to $h_{f}$, there is a sharp gradient in the integrand, resulting in a warning from MATALB when trying to fit the polynomial in the Filon method. This warning is used to trigger the program to switch back to the Gauss method.

The MATLAB program consists of six files in total: (i): The main script. (ii) and (iii): Class definition files where the functions needed for the integration problems in Eqs. (5) and (6) are defined separately. (iv) and (v): Class definition files where the integration procedure using the Gauss and generalized Filon methods are defined, respectively. (vi): The parent class definition file where the input parameters such as densities and wave velocities are defined and passed on to the class definitions (ii) and (iii).

(i) ASM_Adapt_FilonGauss.m
(ii) FunctionsFluid.m
(iii) FunctionsPlate.m
(iv) Integral_Segment_Filon.m
(v) Integral_Segment_Gauss.m
(vi) FunctionsParent.m

**Fig. 1:**
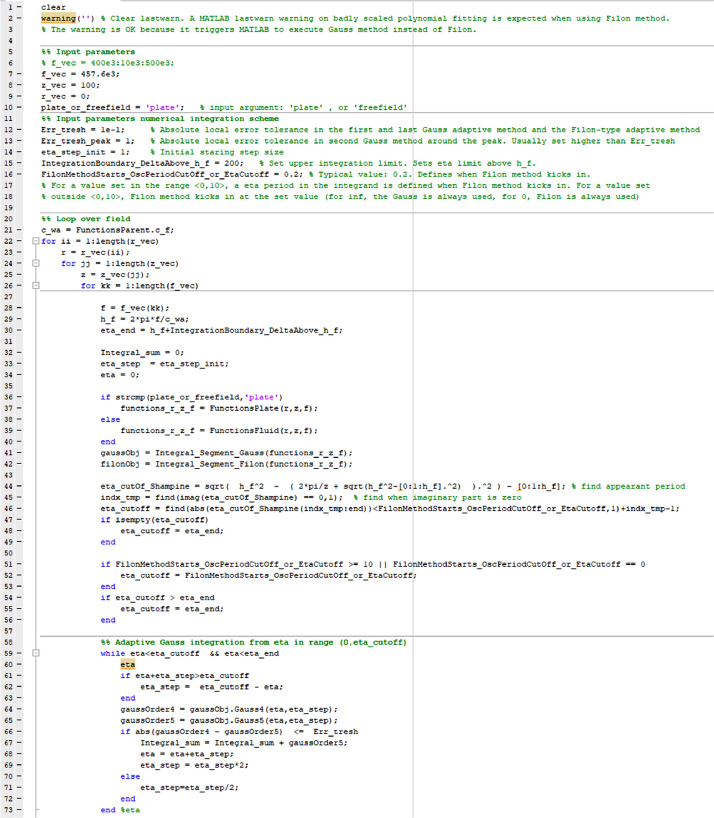
Shows the codelines 1–73 for the script main script (i) ASM_Adapt_FilonGauss.m which calculates the pressure from Eqs. (5) and (6). Input parameters are needed in lines 7–9 and 12–16.

Eqs. (5) and (6) are solved by executing the MATLAB main script (i). To run the program, define the nine input parameters in (i) (lines 7–9 and 12–16 in Fig. 1) and nine input Constant properties in (vi) (lines 5–13 in Fig. 3), then run (i). MATLAB version 2021a has been used in the current work.

**Fig. 2:**
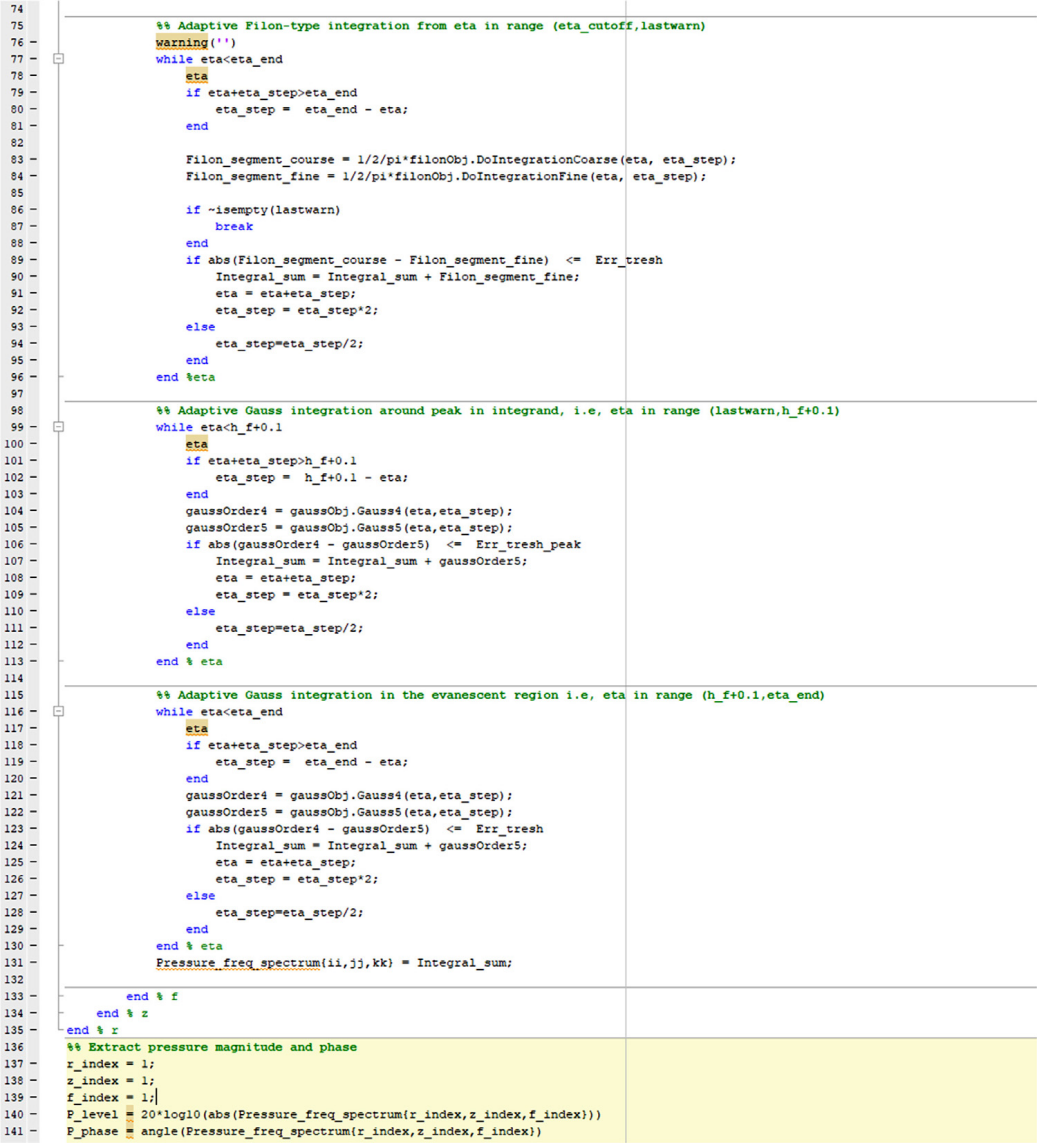
Shows the codelines 74–141 for the script main script (i) ASM_Adapt_FilonGauss.m which calculates the pressure from Eqs. (5) and (6).

The codelines of the main script is presented in Figs. 1 and 2. “f_vec” (line 7) is the frequency vector. “z_vec” (line 8) and “r_vec” (line 9) are vectors defining the z and lateral distances, respectively. “plate_or_freefield” (line 10) must be set to “plate” if the transmitted pressure through a plate is to calculated. Other values calculates the piston pressure freefield without the plate present. “Err_tresh” (line 12) gives the maximum allowed difference between the 4th and 5th order Gauss quadrature rules. It is also the maximum allowed difference between the 3 and 5 point Filon methods. “Err_tresh_peak” (line 13) specifically sets the maximum allowed difference between the 4th and 5th order Gauss quadrature rules for η close to *h_f_*. “eta_step_init” (line 14) is the initial η step. “IntegrationBoundary_DeltaAbove_h_f” (line 15) equals to $\Delta \eta_{cutoff}$ and defines the upper boundary for the integration. “FilonMethodStarts_OscPeriodCutOff_or_EtaCutoff” (line 16) determines when he Filon method is to be used and equals $\Delta \eta_{0}$ when defined between 0 and 10. “FilonMethodStarts_OscPeriodCutOff_or_EtaCutoff” equals to $\eta_{0}$ when the parameter is set to 0 or higher than 10. When set to 0, the Filon method is used over the interval from 0 and close up to *h_f_*.

**Fig. 3:**
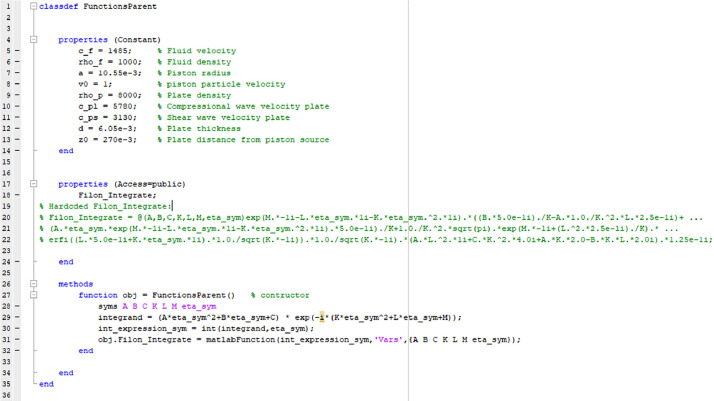
Shows the for the codelines for class definition file (vi) FunctionsParent.m. Input parameters are defined in lines 5–13.

In the script, the final calculated pressure *p*(*r,z,f*) in 131 (Fig. 2) is obtained by looping over the vectors specifying *r, z*, and *f*, respectively. Inside the frequency loop starting at line 26 there are four while loops. In the first, the Gauss method is used (Fig. 1). In the second, the Filon method is used (Fig. 2). In the third and fourth (Fig. 2) the Gauss method is used again. The input parameters of the piston source, fluid and the solid plate are defined in FunctionsParent.m shown in Fig. 3 (lines 5–13).

### Accuracy and computational time

**Fig. 4:**
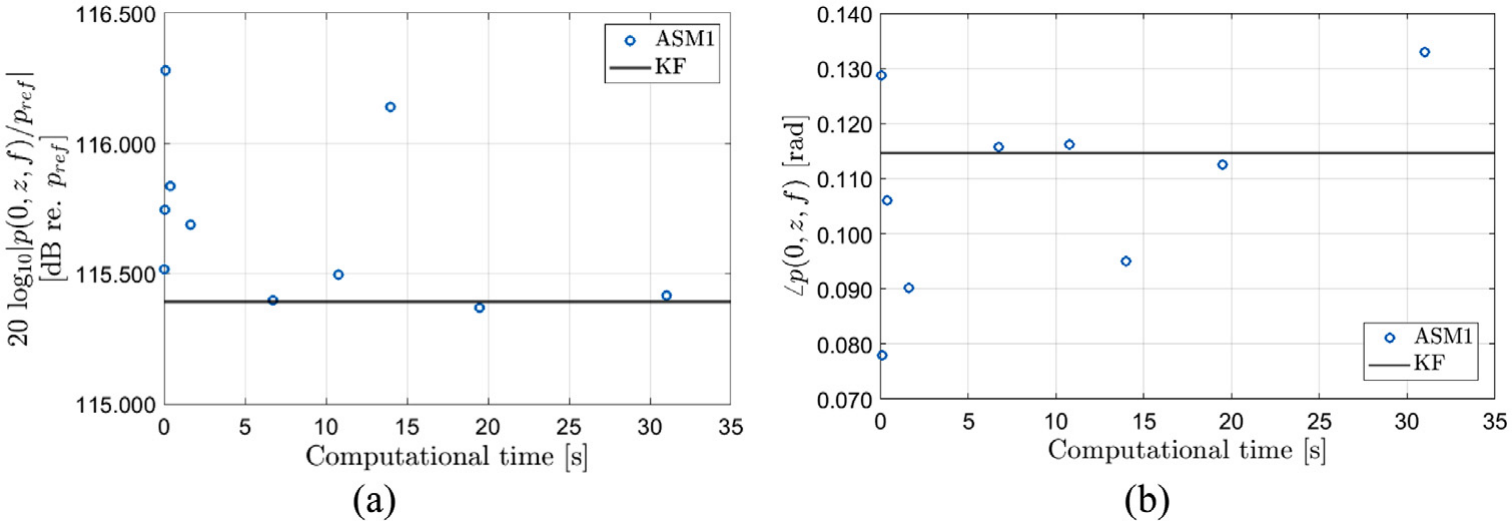
Comparison of calculations of the freefield axial pressure, p(0,z,f), at *z* = 270 mm and f = 457.6 kHz using the traditional ASM1 model, Eq. (1) and the analytical KF model [11]. Wavenumber grid spacings ranging from 16 to 0.5 rad/m is used for the datapoints which corresponds to the different computational times. (a) Magnitude, (b) phase.

In Fig. 4 the axial freefield pressure at *z* = 270 mm and *f* = 457.6 kHz is calculated using the traditional ASM1 method, Eq. (1), with the FFT2 algorithm. The shortest computational time corresponds to a wavenumber grid spacing ($dh_{f,x}$) of 16 rad/m, while the longest corresponds to a grid spacing 0.5 rad/m. The maximum wavenumber in the FFT2 is set to 2136 rad/m for all datapoints (in Figs. 4, 6, and 7) which corresponds to a spatial grid spacing of 1.5 mm (i.e. 0.46 *h_f_*). The solid line is the correct value calculated from the analytical model provided by Kinser et al. [11]. In part (a), the magnitude is shown and in part (b) the phase is shown. Neither the phase nor the magnitude are fully converged.

**Fig. 5:**
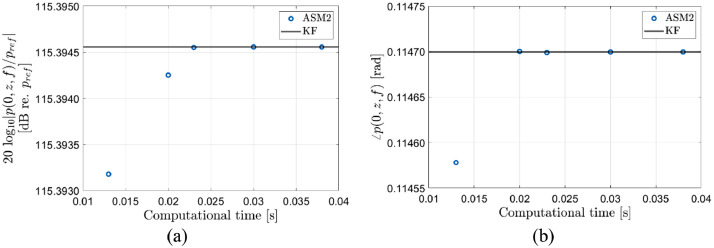
Comparison of calculations of the freefield axial pressure, p(0,z,f), at *z* = 270 mm and *f* = 457.6 kHz using the current ASM2 model, Eq. (6) and the analytical KF model [11]. The computation time depends on the factor “Err_tresh” which is set to 100, 10, 1, 0.1, and 0.01 for the simulations presented here. (a) Magnitude, (b) phase.

In Fig. 5 the axial freefield pressure at *z* = 270 mm and *f* = 457.6 kHz is calculated using the ASM2 method, Eq. (6), with the Gauss/Filon MATLAB program. The integration limit in Eq. (6) is set to 2136 rad/m. The computational times corresponds to “Err_tresh” settings of 100, 10, 1, 0.1, and 0.01. For simplicity, “Err_tresh_peak” is set equal to “Err_tresh”. The solid line is the correct value calculated from the analytical KF model [11]. In part (a), the magnitude is shown and in part (b) the phase is shown. For this example, the accuracy is far better and the computational time is far shorter compared with using the ASM1 method shown in Fig. 4.

**Fig. 6:**
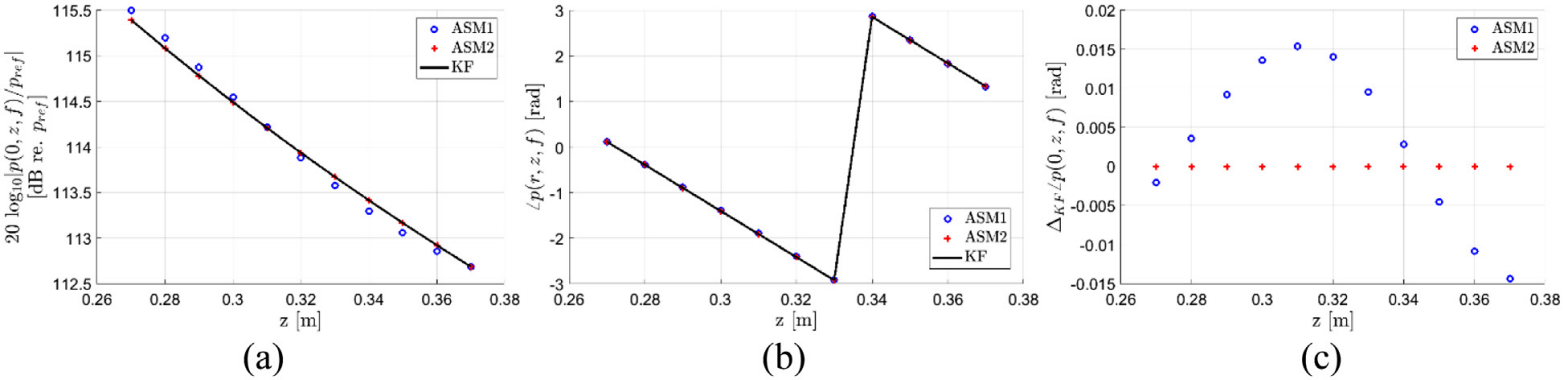
Comparison of calculations of the freefield axial pressure, p(0,z,f), for *z* in the range 270–370 mm and *f* = 457.6 kHz using ASM1, ASM2, and the analytical KF model [11]. In ASM1, the wavenumber grid spacing is 0.6 rad/m. In ASM2, “Err_tresh” is set to 0.1. (a) Magnitude, (b) phase.

In Fig. 6, the axial freefield pressure for *z* in the range 270–370 mm and *f* = 457.6 kHz is calculated using ASM1, ASM2, and the analytical KF model. In ASM1, the wavenumber grid spacing is 0.6 rad/m and the computational time is approximately 50 s. In ASM2, “Err_tresh” is set to 0.1, and the computational time for the datapoints is approximately 1.5 s. For the magnitude in part (a), the maximum deviation between the KF model and ASM1 is approximately 0.1 dB, and 0.000001 dB between the KF model and ASM2. No difference is visually observed in the phase in part (b). In part (c), $\Delta_{KF}$ on the ordinate axis refers to the deviation from the analytical KF model. As can be observed in part (c), there is no visually observable deviation to the ASM2, and a maximum deviation to ASM1 of approximately 0.015 rad/m.

**Fig. 7:**
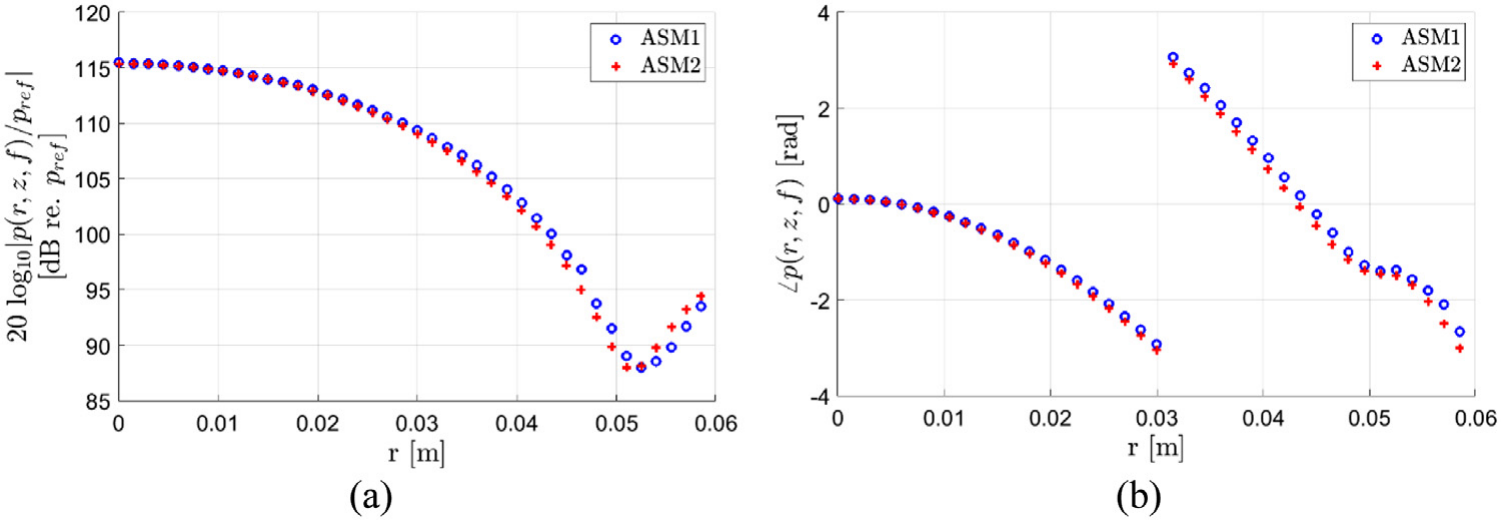
Comparison of calculations of the freefield pressure distribution at *z* = 270 mm for *r* in the range 0–60 mm and *f* = 457.6 kHz using ASM1 and ASM2. In ASM1, the wavenumber grid spacing is 0.6 rad/m. In ASM2, “Err_tresh” is set to 0.1. (a) Magnitude, (b) phase.

In Fig. 7, the freefield pressure distribution at *z* = 270 mm for *r* in the range 0–60 mm and *f* = 457.6 kHz is calculated using ASM1 and ASM2. In ASM1, the wavenumber grid spacing is 0.6 rad/m and the computational time is approximately 20 s. In ASM2, “Err_tresh” is set to 0.1. As no analytical model exists for the pressure distribution for a piston generated nearfield, ASM2 is only compared to ASM1. For ASM2 in Fig. 7, it is assumed that the error is about the same as observed in Figs. 5 and 6. In Fig. 7, the maximum deviation between ASM1 and ASM2 is about 2 dB for the magnitude (a) and about 0.4 rad/m for the phase (b).

## Declaration of Competing Interest

The authors declare that they have no known competing financial interests or personal relationships that could have appeared to influence the work reported in this paper.

## Data Availability

The MATLAB code is attached.

## Appendix Supplementary materials

Image, application 1

Image, application 2

Image, application 3

Image, application 4

Image, application 5

Image, application 6

## References

[1] Johnson R.K., Devaney A.J. (1982). Proceedings of the IEEE International Ultrasonics Symposium.

[2] Whitaker N.A., Haus H.A. (1983). Proceedings of the Ultrasonics Symposium.

[3] Anderson M.J., Martin P.R., Fortunko C.M. (1995). Resonant transmission of a three-dimensional acoustic sound beam through a solid plate in air: theory and measurement. J. Acoust. Soc. Am..

[4] Zeng X., McGough R.J. (2008). Evaluation of the angular spectrum approach for simulations of near-field pressures. J. Acoust. Soc. Am..

[5] Aanes M. (2014).

[6] Waag G., Hoff L., Norli P. (2015). Air-coupled ultrasonic through-transmission thickness measurements of steel plates. Ultrasonics.

[7] Midtbø S.H. (2018).

[8] Sæther M.M., Midtbø S.H., Lunde P. (2022). Interaction of three-dimensional acoustic beam with fluid-loaded solid plate: Axial near- to far-field transmission at normal beam incidence. Ultrasonics.

[9] Evans G., Webster J. (1999). A comparison of some methods for the evaluation of highly oscillatory integrals. J. Comput. Appl. Math..

[10] Shampine L. (2012). Integrating oscillatory functions in MATLAB. Electron. Trans. Numer. Anal..

[11] Kinsler L.E. (2000).

